# Evaluating the sensitivity of EQ-5D in a sample of patients with type 2 diabetes mellitus in two tertiary health care facilities in Nigeria

**DOI:** 10.1186/s13104-015-1832-2

**Published:** 2016-01-12

**Authors:** Obinna Ikechukwu Ekwunife, Charles C. Ezenduka, Bede Emeka Uzoma

**Affiliations:** Cooperative Research Group for Evidence-Based Public Health, Department of Prevention and Evaluation, Leibniz Institute for Prevention Research and Epidemiology—BIPS GmbH, Achterstr. 30, Room 2.120, 28359 Bremen, Germany; Department of Clinical Pharmacy and Pharmacy Management, Faculty of Pharmaceutical Sciences, Nnamdi Azikiwe University, Awka, Anambra State Nigeria

**Keywords:** Validity, Quality of life, EQ VAS, EQ-5D index, Sub-Saharan Africa

## Abstract

**Background:**

The EQ-5D instrument is arguably the most well-known and commonly used generic measure of health status internationally. Although the instrument has been employed in outcomes studies of diabetes mellitus in many countries, it has not yet been used in Nigeria.

**Objective:**

This study was carried out to assess the sensitivity of the EQ-5D instrument in a sample of Nigerian patients with type 2 diabetes mellitus (T2DM).

**Methods:**

A cross-sectional study was conducted using the EQ-5D instrument to assess the self-reported quality of life of patients with T2DM attending two tertiary healthcare facilities in south eastern Nigeria consenting patients completed the questionnaire while waiting to see a doctor. A priori hypotheses were examined using multiple regression analysis to model the relationship between the dependent variables (EQ VAS and EQ-5D Index) and hypothesized independent variables.

**Results:**

A total of 226 patients with T2DM participated in the study. The average age of participants was 57 years (standard deviation 10 years) and 61.1 % were male. The EQ VAS score and EQ-5D index averaged 66.19 (standard deviation 15.42) and 0.78 (standard deviation 0.21) respectively. Number of diabetic complications, number of co-morbidities, patient’s age and being educated predicted EQ VAS score by −6.76, −6.15, −0.22, and 4.51 respectively. Also, number of diabetic complications, number of co-morbidities, patient’s age and being educated predicted EQ-5D index by −0.12, −0.07, −0.003, and 0.06 respectively..

**Conclusion:**

Our findings indicate that the EQ-5D could adequately capture the burden of type 2 diabetes and related complications among Nigerian patients.

## Background

Type 2 diabetes mellitus (T2DM) is a complex and chronic disease that has a significant burden on both the patient and caregivers. A nationally-representative population-based survey among 13,591 Nigerians revealed a diabetes prevalence of 3.3 % [1]. The study also showed that almost half of the population with diabetes (48 %) were unaware about their condition. Furthermore, a higher prevalence of diabetes was associated with urban residence and overweight/obesity [1]. With the continuing urbanisation and the increasing population in Nigeria, the incidence and burden of diabetes is expected to rise further.

The plethora of complications which diabetic patients have to face diminishes their health related quality of life (HRQoL). In addition to diabetes-related complications, episodes and fear of hypoglycaemia, as well as change in lifestyle could further lead to reduction in HRQoL. Thus, from the clinical point of view, HRQoL is an important humanistic outcome measure for assessing diabetic patients’ disease outcome.

Instruments used in assessing HRQoL are generally grouped into preference-based and non-preference-based measures [2]. Whereas preference-based measures assess the value which individuals place on symptoms or quality-of-life states, non-preference-based measures are primarily concerned with directly assessing the symptoms, disease progression, or quality of life [2]. The former combines quality and quantity of life and also incorporate the values that individuals place on particular states of health, thus allowing for estimation of quality-adjusted-life-years (QALYs) [2], a measure commonly used to summarise population health and in the economic evaluation of health interventions and technologies.

The EQ-5D-3L is arguably the most well-known and commonly used generic measure of health status internationally [3]. It is available in 169 languages and has been applied in numerous clinical, cost-effectiveness and population health studies. Of late, it is being used routinely by health-care systems to assess quality of life of patients [3]. Although the instrument has been employed in outcomes studies of diabetes mellitus in many countries [4–12], to our knowledge it has not yet been used in Nigeria. Therefore, the aim of this study was to assess the feasibility of using the EQ-5D-3L in clinical practice and for clinical studies in Nigeria by measuring its sensitivity to disease intensity among Nigerian diabetic patients.

## Methods

### Study design

A cross-sectional study in which the EQ-5D-3L instrument was used to assess the health related quality of life of a sample of patients with T2DM was conducted. The patients were recruited from two teaching hospitals in Anambra state, south east Nigeria, namely: Nnamdi Azikiwe University Teaching Hospital (NAUTH) and Anambra State University Teaching Hospital Amaku (ASUTH). Both hospitals are the only tertiary hospitals in Anambra State, which is one of 36 states in the south-eastern part of Nigeria. Nnamdi Azikiwe University Teaching Hospital (NAUTH) is a 355 bed multi annex complex, providing both specialized and comprehensive medical care services. ASUTH is also a government owned, newly upgraded tertiary health institution, with a 200 bed capacity. Only patients diagnosed with diabetes on the ICD10-E11 i.e., T2DM, and who could read and understand English were included. Severely ill patients as well as those who were in hospital for a follow-up visit were included in the study.

### Study instrument

The EQ-5D instrument, which is described more fully in a separate paper (13), is a standardised instrument for measuring health outcomes. The instrument provides a simple descriptive profile and a single index value for health status. It is primarily designed for self-completion by respondents and is ideally suited for use in postal surveys, in clinics and for face-to-face interviews. It is cognitively simple, taking only a few minutes to complete. The instrument has five dimensions which are mobility; self-care; usual activities; pain/discomfort and anxiety/depression. Each dimension has three levels (no problems, some problems, and major problems) which together define 243 health states (3 to the power of 5 gives the 243 possible combinations). The instrument also has a number of country-specific scoring functions available, which allow for the determination of utilities specific to certain settings. The study adopted a time trade off (TTO) method of health utility measurement as applied in Zimbabwe [13], which is the only African country with country-specific scoring functions. The questionnaire covered the patients’ basic demographic details and clinical information and also included a general health visual analogue scale (EQ VAS). Using a vertical 20 cm graduated line, patients reported their current level of health state by placing a mark on the line. The scale is anchored with 0 to represent the worst imaginable health state, and 100 to represent the best imaginable health state.

### Data administration and collection

Researchers distributed the self-administered questionnaires to the patients while they were waiting to see a doctor in the diabetic clinic of the two tertiary hospitals. Where necessary, the researchers assisted the patients or clarified any queries about the questionnaire. The questionnaires were collected after the patients had completed them. The number of complications (as specified by ICD 10) and co-morbidites (none diabetes mellitus related illness) were afterwards retrieved from clinical notes. The study was conducted between May and August 2014. At NAUTH, this was done on pre-set diabetic clinic days. ASUTH had no special diabetic clinic days and so the survey was conducted on random days.

### Ethical clearance

This study was approved by the Ethics committee of Nnamdi Azikiwe University Teaching Hospital Nnewi, Nigeria. All procedures were carried out according to the study protocol approved by the Ethics Committee. Oral informed consent was obtained from each patient. The information about participants’ identity was not included with the other data and only the principal investigator had access to this information. No reference to the participant’s identity was made at any stage during data analysis.

### Statistical analysis

Health profiles were presented in frequencies stratified by age group. EQ VAS score and EQ-5D index were presented as mean ± standard deviation. In order to establish construct validity, a priori hypotheses on their relevance in T2DM and their ability to test the performance of the EQ VAS score and EQ-5D index were postulated. We hypothesized that (1) patients with more complications will have poor utility, (2) patients with more morbidity will have poor utility, (3) utility will decease with increasing age; and (4) gender will have no effect on the utility of T2DM. The dependent variables were EQ VAS score and EQ-5D index and both were characterised as continuous variables. Independent variables were age, gender, level of education, number of diabetic complications and number of co-morbidities. Age was characterised as continuous variable, gender was treated as binary data while level of education, number of diabetic complication and number of co-morbidities were treated as categorical data. Using the stepwise method, a multiple regression was conducted to examine if the hypothesized independent variables predicted EQ VAS and EQ-5D index. Data analysis was conducted with SPSS 14.0^®^ (Chicago, Illinois, USA). All hypotheses tested were two-tailed, with significance set at *p* < 0.05.

## Results

A total of 226 patients consented to complete the questionnaires used in the study. There were a greater number of male participants as compared to females. Majority of the participants (81 %) formal education compared to those without any formal education. There were more participants with diabetic complications and co-morbidities compared to those without complications and co-morbidity. Details of respondents’ demographic and clinical data are shown in Table 1.

**Table 1:** Demographic and clinical variables of study population and their association with EQ VAS and EQ-5D index (N = 226)

Variable	Frequency (%) or mean ± SD
Gender	
Male	138 (61.1 %)
Female	88 (38.9 %)
Level of education	
No formal education	43 (19.0 %)
Formal education	183 (81.0 %)
Number of diabetic complications	
None	112 (49.6 %)
One	78 (34.5 %)
More than one	36 (15.95)
Number of co-morbidities	
None	98 (43.4 %)
One	93 (41.2 %)
More than one	35 (15.25)
Average age of participants	57 ± 10
Average EQ VAS score of participants	66.19 ± 15.42
Average EQ-5D index of participants	0.78 ± 0.21

Results of multiple regression analysis showed that number of diabetic complications, number of co-morbidities, patients age and being educated predicted EQ VAS score, [*F* (4, 221) = 33.14, *p* = < 0.01, *R*^*2*^ = 0.375]. In the same vein, number of diabetic complications, number of co-morbidities, patients age and being educated predicted EQ-5D index, [*F* (4, 221) = 43.43, *p* = < 0.01, *R*^*2*^ = 0.44]. Details of the multiple regression of EQ VAS score and EQ-5D with demographic and clinical variables are shown in Table 2.

**Table 2:** Multiple regression of EQ VAS score and EQ-5D with demographic and clinical variables (N = 226)

	β	SE	t	*p* value
EQ VAS^a^				
Constant	96.64	5.71	16.94	<0.01
Number of diabetic complications	−6.76	1.22	−5.55	<0.01
Number of co-morbidities	−6.15	1.27	−4.83	<0.01
Age	−0.22	0.09	−2.39	0.02
Patient’s level of education	4.51	2.19	2.06	0.04
Equation-5D index^a^				
Constant	1.25	0.08	16.71	<0.01
Number of diabetic complications	−0.12	0.02	−7.35	<0.01
Number of co-morbidities	−0.07	0.02	−4.43	<0.01
Age	−0.003	0.001	−2.92	<0.01
Patient’s level of education	0.06	0.03	2.10	0.04

^a^Dependent variable

Table 3 shows the health profile of the study population stratified by age group. The percentage of patients with no problem was similar in all health dimensions (>60 %) except for pain/discomfort, where only 47 % of the patients indicated no pain or discomfort.

**Table 3:** Health profile of study population stratified by age group (N = 226)

ED-5D dimension	Age group (frequency)				
	<50	50–59	60–69	>70	Total
Mobility					
No problem	38	55	41	3	137 (61 %)
Some problem	13	18	30	21	82 (36 %)
Confined to bed	0	1	2	4	7 (3 %)
Self-care					
No problem	45	62	54	7	169 (75 %)
Some problem	3	11	16	12	42 (18 %)
Unable to do self-care	2	1	3	9	15 (7 %)
Abilty to perform usual activity					
No problem	39	56	45	4	144 (64 %)
Some problem	11	16	22	13	62 (27 %)
Unable to perform usual activity	1	2	6	11	20 (9 %)
Pain/discomfort					
No pain/discomfort	36	36	33	1	106 (47 %)
Moderate pain/discomfort	13	34	37	24	108 (48 %)
Extreme pain/discomfort	2	4	3	3	12 (5 %)
Anxiety/depression					
Not anxious	39	53	41	11	144 (64 %)
Moderately anxious	11	20	29	14	74 (33 %)
Extremely anxious	1	1	3	3	8 (3 %)

## Discussion

This study assessed the health related quality of life of patients with T2DM using the EQ-5D instrument with the Zimbabwe value set, and predicted the relationship between the dependent variables (EQ VAS and EQ-5D index) and hypothesized independent variable. The presence of diabetic complications, co-morbidities and increasing age were hypothesized to reduce health related quality of life measures (EQ VAS and EQ-5D index) decrease with, while gender was hypothesized to have no influence on EQ VAS and EQ-5D index.

Results of the multiple regression analyses proved the a priori hypothesis to be true. The presence of complications and co-morbidities as well as increasing age resulted in a decrease of EQ VAS and EQ-5D indexes, while gender was not associated with either of the two indexes. Similar results have been shown in other studies. For instance, in their study, Clarke and colleagues observed that higher index scores of 0.1 derived from EQ-5D were associated with an additional 7 % lower risk of vascular events, a 13 % lower risk of complications and up to 14 % lower risk of all-cause mortality [5]. Another study in which symptom severity of diabetic peripheral neuropathy in people with diabetes was correlated with health-related utility revealed that the EQ-5D index fell from an average of 0.81 in those without symptoms to 0.25 in those with severe symptoms [7]. In further studies, diabetic complications and co-morbidities have been shown to have a direct negative effect on HRQoL [12, 14].

In our study, the ED-5D instrument also showed an association between being educated and higher HRQoL (i.e., higher EQ VAS and EQ-5D index). Although no hypothesis on the association between levels of education quality of life was set a priori, the observed finding is logical. Being educated is likely to lead to a better understanding of the disease state and medical interventions required in diabetes management, and thus to greater motivation to self-care and adherence to therapy. Moreover, educated people generally belong to higher socio-economic strata compared to their less educated counterparts and thus can afford better health care services. All these factors can contribute to a higher quality of life. A study of adults conducted in Spain in which the SF-36 Health Survey was used showed similar findings. Perceived health status was observed to decline with decreasing education level, except in women with second level education who had a higher mean rating on various health dimensions than women with third level education [15].

The findings of this study show that EQ-5D is a sensitive instrument for measuring HRQoL outcomes of Nigerian diabetic patients. This is in line with a systematic review on the validity, reliability and responsiveness of the EQ-5D in T2DM studies, which indicated that the EQ-5D can adequately capture the burden of T2DM and related complications in patient subgroups [10]. We therefore postulate that the EQ-5D instrument could be suitable for various applications in Nigeria in relation to diabetic health. These applications may include among others the following: economic evaluations to inform decisions about adoption of new technologies for diabetes care by hospitals, populations health surveys to aid ‘needs based’ allocation of budgets, clinical and observation studies and routine use in hospital to assess providers’ performance.

## Conclusion

The findings of this study indicate that the EQ-5D can adequately capture the burden of type 2 diabetes and related complications in most patient subgroups in Nigeria.

## Abbreviations

EQ-5D VAS: visual analogue scale, of standard layout, for recording an individual’s rating of EQ-5D health states
EQ-5D VAS score: score recorded by an individual for an EQ-5D health state on the EQ-5D VAS
EQ-5D Index: index attached to the EQ-5D state according to a particular set of weights

## References

[1] Kyari F, Tafida A, Sivasubramaniam S, Murthy GV, Peto T, Gilbert CE (2014). Prevalence and risk factors for diabetes and diabetic retinopathy: results from the Nigeria national blindness and visual impairment survey. BMC Public Health.

[2] Gray AMCP, Wolstenholme JL, Wordsworth S (2011). Applied methods of cost-effectiveness analysis in health care.

[3] Devlin NJ, Krabbe PF (2013). The development of new research methods for the valuation of EQ-5D-5L. Eur J Health Econ.

[4] Quality of life in type 2 diabetic patients is affected by complications but not by intensive policies to improve blood glucose or blood pressure control (UKPDS 37). UK prospective diabetes study group. Diabetes Care. 1999;22(7):1125–36. 10.2337/diacare.22.7.1125 10388978

[5] Clarke PM, Hayes AJ, Glasziou PG, Scott R, Simes J, Keech AC (2009). Using the EQ-5D index score as a predictor of outcomes in patients with type 2 diabetes. Med Care.

[6] Currie CJ, Morgan CL, Poole CD, Sharplin P, Lammert M, McEwan P (2006). Multivariate models of health-related utility and the fear of hypoglycaemia in people with diabetes. Curr Med Res Opin.

[7] Currie CJ, Poole CD, Woehl A, Morgan CL, Cawley S, Rousculp MD (2006). The health-related utility and health-related quality of life of hospital-treated subjects with type 1 or type 2 diabetes with particular reference to differing severity of peripheral neuropathy. Diabetologia.

[8] Grandy S, Fox KM (2012). Change in health status (EQ-5D) over 5 years among individuals with and without type 2 diabetes mellitus in the SHIELD longitudinal study. Health Qual Life Outcomes.

[9] Grandy S, Langkilde AM, Sugg JE, Parikh S, Sjostrom CD (2014). Health-related quality of life (EQ-5D) among type 2 diabetes mellitus patients treated with dapagliflozin over 2 years. Int J Clin Pract.

[10] Janssen MF, Lubetkin EI, Sekhobo JP, Pickard AS (2011). The use of the EQ-5D preference-based health status measure in adults with type 2 diabetes mellitus. Diabet Med.

[11] Morgan CL, McEwan P, Morrissey M, Peters JR, Poole C, Currie CJ (2006). Characterization and comparison of health-related utility in people with diabetes with various single and multiple vascular complications. Diabet Med.

[12] Sakamaki H, Ikeda S, Ikegami N, Uchigata Y, Iwamoto Y, Origasa H (2006). Measurement of HRQL using EQ-5D in patients with type 2 diabetes mellitus in Japan. Value Health.

[13] Jelsma J, Hansen K, De Weerdt W, De Cock P, Kind P (2003). How do Zimbabweans value health states?. Popul Health Metr.

[14] Hayes AJ, Clarke PM, Glasziou PG, Simes RJ, Drury PL, Keech AC (2008). Can self-rated health scores be used for risk prediction in patients with type 2 diabetes?. Diabetes Care.

[15] Regidor E, Barrio G, de la Fuente L, Domingo A, Rodriguez C, Alonso J (1999). Association between educational level and health related quality of life in Spanish adults. J Epidemiol Community Health.

